# Deaths from Nervous Diseases

**Published:** 1876-04

**Authors:** 


Deaths from Nervous Diseases.—In an article by Dr. Althaus, in the *British Medical Journal*, one point considered by the author was the proportion of deaths from delirium tremens in the different divisions of England and Wales. It was found that London headed the list with 100, the southeastern counties followed with 62, the northwestern with 57, the south midland with 55, the northern with 54, Yorkshire with 42, the eastern division with 41, the west midland with 40, the southwestern with 39, the north midland with 36, and last of all came Wales, with only 27. Dr. Althaus thought it very significant that London, where nervous diseases were at a comparatively low ebb, should consume proportionately so much more alcohol than Wales, where these maladies were singularly rife; and he asked whether a large consumption of strong alcoholic drinks was really always prejudicial for the nervous system, as had perhaps been too sweepingly asserted by many well-intentioned persons of late years. The author next considered the prevalence of these diseases during life as distinguished from their mortality, and remarked that on this point we had definite information only with regard to one disease, viz : insanity, in the Reports of the Commissioners in Lunacy. From these it appeared that there were eighty-eight lunatics living to one death from insanity registered by the Registrar-General; and that of five lunatics who died, only one died from insanity, and the other four from different diseases. He thought the prevalence of cephalitis only slightly higher than its mortality; of paralysis, he reckoned the living patients as twelve to one death; of apoplexy, nothing definite was known in this respect; chorea was fatal in one out of a hundred cases actually occurring; in delirum tremens, three out of four patients recovered, which would give four cases to each death; in tetanus, the proportion was two deaths to three cases; in epilepsy, which was but rarely fatal, he reckoned fifty living sufferers to one death; of convulsions, it was difficult to speak definitely, as the death-rate from them was in the process of changing considerably. Hysteria was very prevalent, but scarcely ever fatal. Dr. Althaus concluded his paper with some remarks on the probability of therapeutics becoming gradually able to influence the mortality from nervous diseases. He saw good reason to believe that convulsive disorders would undergo a further considerable diminution under the influence of improved treatment. Not only for epilepsy and eclampsia, but also for tetanus, recent researches seemed to hold out a prospect of these diseases becoming eventually more amenable to treatment; while chorea was known to kill but rarely. Paralysis, apoplexy, and insanity, however, would no doubt always carry off large numbers of victims as long as the present conditions of our existence, with its passions and sorrows, its troubles and disappointments, continued.

The following bit of raillery from Joseph Addison would be very distasteful to the medical profession, were it not the case that it was written in the greatest good humor:

"If we look into the profession of physic we shall find a most formidable body of men. The sight of them is enough to make a man serious; for we may lay it down as a maxim that when a nation abounds in physicians it grows thin of people. This body of men in our own country may be described like the British army in Cæsar's time: some of them slay in chariots and some on foot. If the infantry do less execution than the charioteers it is because they can not be carried into all quarters of the town and dispatch so much business in so short a time. Besides this body of regular troops, there are stragglers who, without being duly listed and enrolled, do infinite mischief to those who are so unlucky as to fall into their hands. There are, besides the above-mentioned, innumerable retainers to physic who, for want of other patients, amuse themselves with the stifling of cats in an air-pump, cutting up dogs alive, or impaling of insects upon the point of a needle for microscopical observation, besides those that are employed in the gathering of weeds and the chase of butterflies.—*Louisville Medical News.*

